# Nutritional Status Indicators as a Predictor of Achieving Remission at Week 14 during Vedolizumab Therapy in Patients with Ulcerative Colitis: A Pilot Study

**DOI:** 10.3390/nu15010240

**Published:** 2023-01-03

**Authors:** Aleksandra Sobolewska-Włodarczyk, Ewa Walecka-Kapica, Marcin Włodarczyk, Anita Gąsiorowska

**Affiliations:** 1 Department of Gastroenterology, Medical University of Lodz, Pomorska Str. 251, 90-213 Lodz, Poland; 2 Department of General and Oncological Surgery, Medical University of Lodz, Pomorska Str. 251, 90-213 Lodz, Poland

**Keywords:** ulcerative colitis, vedolizumab, nutritional status, predictive biomarkers, treatment outcomes

## Abstract

**Background**: The loss of response or failure to achieve remission to vedolizumab in ulcerative colitis (UC) patients is currently a major clinical problem. Recently, Nutritional Risk Index (NRI), Controlling Nutritional Status (CONUT), and Malnutrition Universal Screening Tool (MUST) have been suggested as a new prognostic factor of UC activity. Here, we aimed at confirmation of hypotezis that NRI, CONUT and MUST may be used as inexpensive and efficient predictive biomarkers of response in UC patients treated with vedolizumab. **Methods**: This study was conducted in retrospective manner in 32 adult patients with UC of Caucasian origin (21 men and 11 women), who were qualified for 52-week therapy with vedolizumab and finished the 14-weeks from January 2020 to March 2022. Our study analyzed the 45 courses of vedolizumab therapy. Nutritional status indicators, i.e., the NRI, CONUT and MUST of each UC patient, were marked at the time of qualifying for biological treatment. **Results**: In our study, the MUST score was significantly lower in UC patients who positively achieved clinical remission at week 14 during vedolizumab induction therapy (0.33 ± 0.49 vs. 1.37 ± 0.83; *p* = 0.002). The analysis showed the lower baseline NRI and CONUT scores in patients with positive clinical remission at week 14 (NRI: 96.42 ± 4.29 vs. 101.41 ± 7.09; *p* = 0.024; CONUT: 1.00 ± 1.08 vs. 2.16 ± 1.46; *p* = 0.031). **Conclusions**: Nutritional status indicators (NRI, MUST and CONUT) may become valuable predictor of achieving remission at week 14 during vedolizumab therapy in UC patients.

## 1. Introduction

Ulcerative colitis (UC) is a chronic condition that results in the inflammation of the colon and rectum. The primary symptoms of exacerbation of the disease are abdominal pain and bloody diarrhea. The etiopathology of UC remains not fully recognized [1]. Current theories involve the impact of immune system dysfunction, environmental factors, genetics, and changes in the physiological gut microbiota. The incidence rates tended to be higher in developed countries with some theories suggesting this to be the result of environmental factors, especially the “Westernization” of lifestyle, less exposure to intestinal infections and the change of diet to a “Western diet”—rich in protein and saturated fatty acids, low in vegetables, fruit and other high-fiber foods, as well as with a broad use of food additives [2,3,4]. Diagnosis is typically by colonoscopy with tissue biopsies [5]. The treatment of inflammatory bowel diseases is a lifelong therapy. Currently, the drug that will cure the patient is unknown. The aim of the current therapies is to achieve clinical and endoscopic remission. There is strong evidence showing that monoclonal antibodies are effective in moderate to severe ulcerative colitis refractory to conventional therapy. Vedolizumab is currently well established in the treatment of both Crohn’s disease (CD) and ulcerative colitis. Vedolizumab is a humanized monoclonal antibody, which blocking the α_4_β_7_ integrin results in gut-selective anti-inflammatory activity [6]. In UC patients, this anti-integrin drug was proven to be effective and safe agent in the management of moderate-to-severe exacerbation in recent randomized clinical trials [7]. This study aimed to identify the risk factor for the exacerbation of inflammatory bowel diseases (IBD), as well as the predictors of response to treatment. Nutritional deficiency is a common complication of long-term IBD. In order to objectify nutritional disorders in everyday practice, scales such as NRS-2002 (Nutritional Risk Score) or Subjective Global Assessment (SGA) are used. Objectivized nutritional indicators are becoming a more and more frequently used tool in clinical work. It is speculated that malnourished and overweight patients may respond worse to the treatment of IBD patients. Identifying risk factors for non-response to treatment may be very useful in targeting the best treatment approach.

## 2. Methods

### 2.1. Patients

This retrospective clinical study was performed in 32 adult patients 18–70 years of age with UC of Caucasian origin (11 women and 21 men), who were included for 52-week therapy with vedolizumab and finished the 14-week of treatment. The enrolment of patients was performed in dates from January 2020 to March 2022. Our study analyzed the 45 courses of vedolizumab therapy. UC was diagnosed and confirmed according to clinical, endoscopic, radiological, and histological guidelines published by the European Crohn’s and Colitis Organization (ECCO) [5]. At the designated time points, the clinical status of disease in each patient was classified according to the total Mayo score, which is a complex scoring system based on four parts: physician assessment, stool frequency, rectal bleeding, and endoscopy. Each parameter is rated from 0 to 3, giving a total score of 0 to 12. A summary score of 3 to 5 points indicates mildly active disease, a score of 6 to 10 points indicates moderately active disease, and a score of 11 to 12 points indicates the severe exacerbation of UC.

The enrollment criteria for vedolizumab therapy involved moderate-to-severe exacerbation of UC and the ineffectiveness of previously used non-biological therapies, such as mesalazine, immunomodulators and corticosteroids. All patients qualified for this study had luminal activity of UC with ulcers, confirmed in colonoscopy just prior to initiation of vedolizumab therapy. All patients enrolled to vedolizumab therapy received induction treatment with a 300 mg vedolizumab intravenous infusion at baseline and further infusions at dose 300 mg after 2 and 6 weeks.

Response to vedolizumab therapy was defined as a decrease in disease activity of at least 3 total Mayo points at week 14. Nutritional status indicators, i.e., BMI (*Body Mass Index*), NRI (*Nutritional Risk Index*), CONUT (*Controlling Nutritional Status*) and MUST (*Malnutrition Universal Screening Tool*) of each UC patient were marked at the time of qualifying for biological treatment. The clinical assessment of all enrolled patient was performed at each visit related to the drug administration at 0, 2, 6 and 14 weeks of treatment.

From this study, according to the NDP (National Drug Program) and local Summary of Product Characteristics, were excluded UC patients with: hyperreactivity to vedolizumab or excipients; precancerous condition or malignancy diagnosed within 5 years prior to study enrollment; chronic heart, kidney, liver, or respiratory failure; demyelinating disease; severe active or opportunistic infections (e.g., progressive multifocal leukoencephalopathy); pregnancy or breastfeeding.

At inclusion in the study (before first vedolizumab infusion) BMI was calculated based on the height and weight measurements in each patient: BMI = weight in kilograms/height in m^2^. Normal range was considered 20–25 kg/m^2^, obesity > 30 kg/m^2^, overweight 20–25 kg/m^2^, borderline underweight 18.5–20 kg/m^2^, undernutrition <18.5 kg/m^2^.

At inclusion in the study also NRI was calculated as follows:

NRI = [1.519 × serum albumin (g/L)] + [ 41.7 × (current/usual body weight)].

Patients with an NRI > 97.5 were considered not at risk, patients with NRI between 83.5 and 97.5 were considered moderately at risk and those patients with NRI < 83.5 were considered having severe risk by NRI.

CONUT score was evaluated from serum albumin concentration, triglyceride level and total lymphocyte count.

Patients were separated into three groups according to the CONUT score: normal (0–1), mild (2–4), moderate (5–8) and severe (9–12).

MUST involved the assessment of three main parameters: patient BMI, unintentional weight loss in the past 3–6 months, and acute disease effect, implying a patient that is acutely ill and there has been or is likely to be no nutritional intake for >5 days. The first two parameters receive 0, 1 or 2 points each, and the last parameter receives 2 points in case of positivity. A total score of 0, 1 and ≥2 denotes low, medium and high risk for malnutrition, respectively.

### 2.2. Collection of Blood Samples and Blood Analysis

From all patients, 2 mL venous blood was taken into standardized tubes containing ethylenediaminetetraacetic acid (EDTA) to determine C-reaction protein (CRP), Ht (hematocrit), white blood cell count (WBC), serum albumin concentration, triglyceride level and total Lymphocyte count. In UC patients, blood samples for analysis were obtained before initiation of 52-week vedolizumab. Blood analysis was performed within 2 h after collection using the same automatic analyzer. The adult normal reference range for WBC 4.5–10.3 × 10^9^/L. Also, 2 mL blood samples were collected into serum tube and CRP was determined using automatic devices (adult normal reference range for CRP: <0.5 mg/dL).

### 2.3. Statistical Analysis

The analysis of data collected in the study has been performed with the statistical package Statistica 13.1 (StatSoft, Inc., 2300 E 14th Tulsa, OK, USA). The analyzed results have been presented as a mean standard deviation regarding continuous variables and as numbers and percentage referring to categorical variables. The estimation of normality of distribution of the examined quantitative parameters has been executed with the W Shapiro–Wilk test. The comparisons of the study groups have been performed with the Student’s *t*-test (or nonparametric the Mann–Whitney test, depending on the distribution of variables) and the chi-squared test (or Fischer test). In all the analyses the probability value *p* < 0.05 has been considered statistically significant.

## 3. Results

A total sample of 32 patients who underwent 14-week vedolizumab induction therapy from January 2020 to March 2022 at the Department of Gastroenterology were enrolled in our study: 21 men (65.6%) and 11 women (34.4%). The most common location of inflammatory lesions was the left colon (*n* = 18), next pancolitis (*n* = 12) and rectum (*n* = 1). The average BMI of patients was 25.37 kg/m^2^. Undernutrition < 18.5 kg/m^2^ was reported in 3 patients (*n* = 3), and obesity > 30 kg/m^2^ in 8 patients (*n* = 8). The mean duration of the disease was 7.03 years. In our study, nearly 41% (*n* = 13) of the UC patients responded positively to the 14-week induction therapy. The study group involved 31.3% (*n* = 10) patients bio-naïve to previous biological therapies. The sociodemographic and baseline characteristics data of all patients enrolled in our study are presented in Table 1. 

There was no relationship between the baseline state of disease in total Mayo score and the positive response to 14-week vedolizumab induction therapy (9.81 ± 1.05 vs. 9.74 ± 1.19; *p* = 0.868). Our study showed that patient naïve to previous biological therapies significantly more often achieved clinical remission at week 14 during vedolizumab induction therapy (40.4% vs. 70.0%; *p* = 0.035) (Table 1). In all UC patients, there was no significant association between clinical remission at week 14 and gender (*p* = 0.687) or age (37.0 yeas ± 16.2 yeas vs. 38.5 yeas ± 15.1 yeas; *p* = 0.585) (Table 1). The analysis showed no significant relationship between response to vedolizumab at week 14 and colonic locations of inflammatory lesions (*p* = 0.364), baseline BMI (25.79 ± 5.30 vs. 24.99 ± 5.18; *p* = 0.808), and disease duration (7.0 yeas ± 4.1 yeas vs. 7.1 yeas ± 3.4 yeas; *p* = 0.966) (Table 1). Laboratory tests revealed no significant relationships between clinical remission at week 14 and CRP (17.71 ± 29.71 vs. 7.43 ± 6.24; *p* = 0.307), WBC (9.56 ± 3.09 vs. 8.58 ± 2.89; *p* = 0.994) and hemoglobin levels (13.00 ± 1.62 vs. 12.53 ± 2.05; *p* = 0.509).

**Figure 1:**
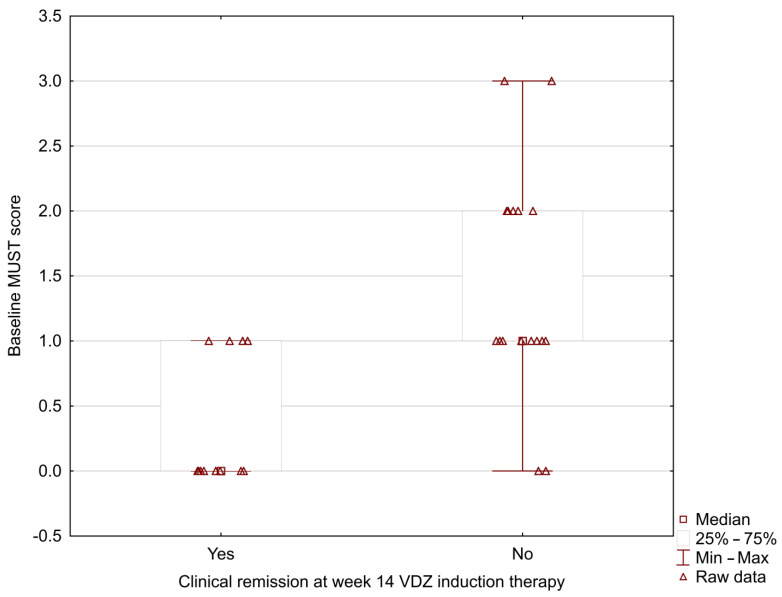
Relationship between the baseline MUST score and clinical remission at week 14 during vedolizumab induction therapy (0.33 ± 0.49 vs. 1.37 ± 0.83; *p* = 0.002).

**Figure 2:**
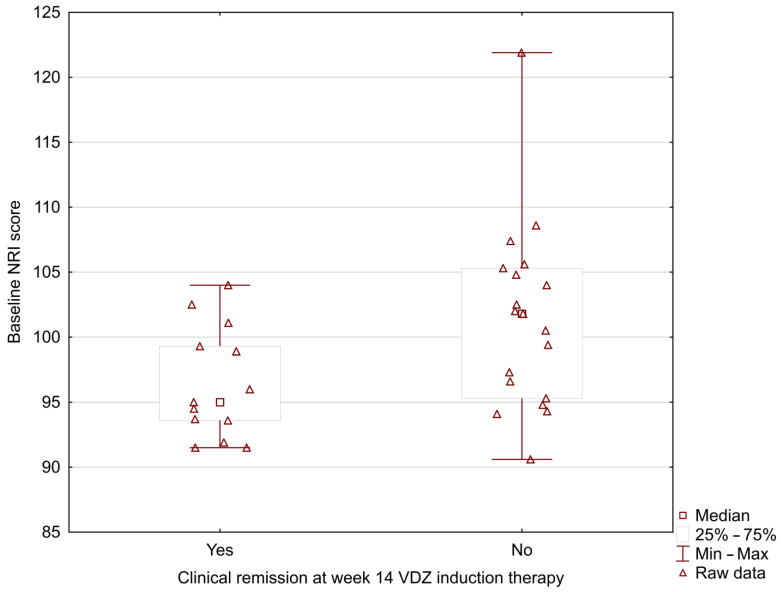
Relationship between the baseline NRI score and clinical remission at week 14 during vedolizumab induction therapy (96.42 ± 4.29 vs. 101.41 ± 7.09; *p* = 0.024).

**Figure 3:**
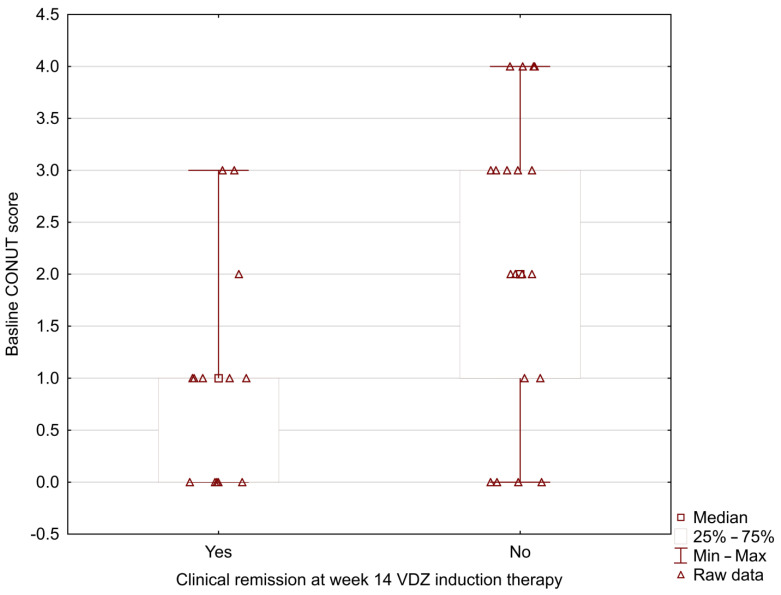
Relationship between the baseline CONUT score and clinical remission at week 14 during vedolizumab induction therapy (CONUT: 1.00 ± 1.08 vs. 2.16 ± 1.46; *p* = 0.031).

In our study, the MUST score was significantly lower in UC patients who positively achieved clinical remission at week 14 during vedolizumab induction therapy (0.33 ± 0.49 vs. 1.37 ± 0.83; *p* = 0.002) (Figure 1). The analysis showed the lower baseline NRI and CONUT scores in patients with positive clinical remission at week 14 (NRI: 96.42 ± 4.29 vs. 101.41 ± 7.09; *p* = 0.024; CONUT: 1.00 ± 1.08 vs. 2.16 ± 1.46; *p* = 0.031) (Figure 2 and Figure 3).

## 4. Discussion

We observed that nutritional status indicators may be good predictors of positive response to vedolizumab therapy in UC patients. Observation of NRI, MUST and CONUT during treatment, especially before the first administration of vedolizumab, may become a useful tool in personalized therapy and predicting outcomes of UC treatment.

Vedolizumab, which is widely used in UC patients, effectively controls clinical symptoms, maintains remission, prevents relapses, improves quality of life, and reduces mortality [8]. Furthermore, most UC patients respond positively to infliximab and clinical response after induction of remission is often achieved. Nevertheless, the lack of response in UC patients during initial treatment with vedolizumab is still a common problem. Therefore, a predictor of response to anti-integrin therapy is essential in clinical practice [9].

The treatment of patients with UC is long-term and is often associated with the failure of previous therapy. Treatment with vedolizumab has been used as the first-line treatment in biological therapy for several years [10]. However, many patients have already received biological treatment in the past. Our study showed that patients naïve to previous biological therapies significantly more often achieved clinical remission at week 14 during vedolizumab induction therapy (70.0% vs. 27.3%; *p* = 0.023). The same results were observed in POLONEZ study performed by Cichoż-Lach et al. This study, conducted prospectively, included adult UC patients eligible for UC treatment with vedolizumab who were recruited from 12 centers in Poland and showed that among biologic-exposed patients (mostly infliximab-treated), 57% had failed to respond to the therapy [11].

Nutritional status is a very important aspect of treating patients. Individuals in exacerbation have a much higher energy expenditure. There is more and more emphasis on the objectification of the nutritional status. In clinical practice, indicators such as BMI, NRI are commonly used, while CONUT and MUST are mainly abused in scientific work. The search for predictors of response to treatment is the goal of current research. It has been speculated that malnutrition may be a factor in non-response to treatment.

One of the tools used to identify patients with different nutritional status is CONUT (Controlling Nutritional Status) [12]. The first time this tool was validated was by Ulíbarri et al. in 2005 [12]. There are not many reports in the literature on scales of evaluation of malnutrition in UC patients. There are reports on the effectiveness in various other diseases, including oncologic [13,14,15,16]. Kheirouri et al., in 2021, showed that a high preoperative CONUT score is associated with poor survival rate and is an independent prognostic factor of overall survival and cancer-specific survival in patients with various types of cancer [17]. Researchers suggested that the evaluation of the preoperative CONUT score might help clinicians in decision-making with respect to surgical implications. Only J L de-León-Rendón et al. showed that patients with a high (>6 points) CONUT score presented with moderate-to-severe activity on the Truelove and Witts scale. [18] Researchers speculated that the CONUT score could be a promising tool for evaluating nutritional status in UC patients and predicting UC severity. In our study, we showed the lower baseline CONUT scores in patients with positive clinical remission at week 14 (1.00 ± 1.08 vs. 2.16 ± 1.46; *p* = 0.031). Nutritional status indicators are also used for CD. Patients often require surgery, and the postoperative course affects their quality of life. In 2020, Dong et al. showed that in CD patients a preoperative CONUT score cut-off value of more than 3.5 could may help in identifying the patient with a high possibility of malnutrition and postoperative complications [19]. Researchers presented a hypothesis that the preoperative CONUT score was an independent risk factor for complications (OR 3.507, 95% CI 1.522–8.079, *p* = 0.003). In addition, in their study, postoperative complications were correlated with BMI, preoperative albumin, the preoperative CONUT score, and preoperative infliximab use. 

The next tool used was MUST—the Malnutrition Universal Screening Tool. MUST was developed by the Malnutrition Advisory Group, a committee of British Association of Parenteral and Enteral Nutrition (BAPEN) in 2003. It has been used since then as a very useful screening tool in hospitals in many countries of the world. In our study, the MUST score was significantly lower in UC patients who positively achieved clinical remission at week 14 during vedolizumab induction therapy. Currently, there are no papers describing the use of this insert in biologically treated patients. However, the interest in its use is growing. Keetarut et al. suggested that self-screening using MUST could be effectively used in an IBD outpatient clinic to identify those at medium and high risk of malnutrition [20]. The patient friendly version of MUST (‘MUST’-P) was considered quick and easy to use by patients. The implementation of self-screening with MUST could improve the nutritional management of IBD patients. The ‘MUST’-P was the MUST tool developed by Cawood et al., who adapted MUST for patient use in a hospital outpatient setting [21]. The BMI and weight loss charts were used from the British Association for Parenteral and Enteral Nutrition (BAPEN) tool kit [22]. Following completion of the ‘MUST’-P, the patient was asked to rate the ease-of-use of the ‘MUST’-P tool on a Likert scale (very difficult to very easy) and time for completion (in minutes) was estimated by the patient.

The use of malnutrition indicators should be important in clinical practice, especially in patients with IBD. An attempt to develop indicators suitable for this group is the subject of interest of the current research. The valuable work was published in 2021 by researchers from Israel aimed to identify IBD-related risk factors for development of malnutrition. Einav et al conducted retrospective matched case-control study to identify IBD-related risk factors for development of malnutrition [23]. They proved that independent IBD-related malnutrition risk factors were: 18.5 ≤ BMI ≤ 22 kg/m^2^, high annual healthcare utility and endoscopic disease activity. The IBD-MR was positively associated with malnutrition development independently of the MUST score. In their study among patients with low MUST scores determined during the index visit, identification of ≥2 IBD-MR factors was strongly associated with malnutrition development. Avoiding the development of malnutrition in a patient can protect him from serious complications of the disease.

In our study, we did not observe any relationship between body mass index and efficacy of biological treatment. This is in line with Farraye et al., who concluded that efficacy the tofacitinib therapy in patients with UC was similar regardless of BMI [24]. Contrary, Kurnool et al. evaluated the impact of obesity on response to biologic therapy in patients with UC [25]. They concluded that high BMI is independently associated with increased risk of treatment failure in biologic-treated patients with UC, independent of dosing regimen [26].

In IBD, vedolizumab is administered as a non-weight-based fixed dose. It is speculated that higher BMI is associated with lower serum vedolizumab levels, but it is unclear whether it is associated with an unfavorable response to vedolizumab. For this reason, Levine et al. published their study. The researchers showed that obesity (BMI ≥ 30 kg/m > 2) was not associated with higher rates of vedolizumab dose escalation. However, it was associated with lower rates of vedolizumab discontinuation and CRP normalization, but not steroid-free clinical remission or endoscopic remission [27].

Treatment failure and IBD-related surgery is a important problem in clinical practice. The recognition of modifiable risk factors for therapy failure would allow individualization of therapy. In 2018, Kurnool et al. conducted a study to evaluate the impact of obesity on real world response to biological therapy in patients with UC. They proved that high BMI is independently associated with increased risk of treatment failure, including IBD-related surgery or hospitalization, and may be a lower risk of achieving endoscopic remission. [25].

Elderly patients with UC are an increasing clinical challenge. Due to the progress of treatment, patients over 60 years of age constitute an increasing population of IBD patients. Higashiyama et al. suggested that the risks of hospitalization and surgery were elevated as age advanced [28]. Moreover, UC is diagnosed more and more often in the elderly. Nutritional disorders are more common in this group of patients. A valuable research, describing the nutritional issues and treatment failure in UC patients, was published in 2021. In their study, the value of geriatric nutritional risk index negatively correlated with disease activity, could distinguish severe activity and discriminate the elderly-onset UC patients suffering from surgery and hospitalization. Researchers proposed that malnutrition estimated by geriatric nutritional risk index was significantly related with poor clinical courses of the elderly-onset UC patients, suggesting that the evaluation of nutritional status at the onset might be useful for predicting risks of clinical courses [28].

## 5. Conclusions

Our study showed that nutritional status indicators (NRI, CONUT, MUST) may be a useful biomarker of achieving remission at week 14 during vedolizumab therapy in UC patients. We suggest that nutritional status indicators, as easily available prognostic factors, will allow clinicians to individualize the treatment to achieve the best clinical outcomes. Perhaps, improvement in nutritional status prior to biological therapy may be needed in achieving steroid-free clinical remission. However, this study was performed on a small group of UC patients and further research are warranted to confirm our observations on the predicting role of nutritional status and to establish the cutoff points in a larger cohort. Additionally, the possible application of this parameter in UC patients treated with other biological agents needs verification.

## Figures and Tables

**Table 1:** Baseline clinical characteristics in the ulcerative colitis (UC) patients with and without achievement of clinical remission after 14 weeks of vedolizumab induction therapy.

		Clinical Remission after 14 Weeks of VDZ		p *
		Yes	No	
Subjects, *n*		13	19	NA
**Sex**	women, *n* (%)	5 (38.5%)	6 (31.6%)	0.687
	men, *n* (%)	8 (61.5%)	13 (68.4%)	
Age, y		37.0 ± 16.2	38.5 ± 15.1	0.585
**BMI, kg/m^2^**		25.79 ± 5.30	24.99 ± 5.18	0.808
**Location of inflammatory lesions**	pancolitis, *n* (%)	6 (50.0%)	6 (31.6%)	0.473
	left colon, *n* (%)	6 (50.0%)	12 (63.2%)	
	rectum, *n* (%)	0 (0.0%)	1 (5.2%)	
**Mayo score**		9.81 ± 1.05	9.74 ± 1.19	0.868
**Bio-naïve**	Yes, *n* (%)	6 (46.2%)	16 (84.2%) 3 (15.8%)	**0.023**
	No, *n* (%)	7 (53.8%)		
Disease duration, y		7.0 ± 4.1	7.1 ± 3.4	0.966
Hemoglobin, g/dL		13.00 ± 1.62	12.53 ± 2.05	0.509
White blood cell, ×10^3^/μL		9.56 ± 3.09	8.58 ± 2.89	0.994
CRP, ×10^3^/μL		17.71 ± 29.71	7.43 ± 6.24	0.307

Data are presented as mean ± standard deviation or number (percentage). *p* *—statistical significance between groups with and without clinical remission after 14 weeks of vedolizumab induction therapy BMI-body mass index; NA—not applicable; VDZ—vedolizumab.

## Data Availability

The datasets used and/or analyzed within the framework of this study are available from the corresponding author on reasonable request.
